# Multicentre, randomised controlled trial of physiological-based cord clamping versus immediate cord clamping in infants with a congenital diaphragmatic hernia (PinC): statistical analysis plan

**DOI:** 10.1186/s13063-024-08027-7

**Published:** 2024-03-20

**Authors:** Emily J. J. Horn-Oudshoorn, Marijn J. Vermeulen, Ronny Knol, Rebekka Bout-Rebel, Arjan B. te Pas, Stuart B. Hooper, Suzan C. M. Cochius-den Otter, Rene M. H. Wijnen, Kelly J. Crossley, Neysan Rafat, Thomas Schaible, Willem P. de Boode, Anne Debeer, Berndt Urlesberger, Calum T. Roberts, Florian Kipfmueller, Irma Capolupo, Carmen M. Burgos, Bettina E. Hansen, Irwin K. M. Reiss, Philip L. J. DeKoninck

**Affiliations:** 1 Division of Neonatology, Department of Neonatal and Paediatric Intensive Care, Erasmus MC University Medical Center, Rotterdam, the Netherlands; 2 Division of Neonatology, Department of Paediatrics, Leiden University Medical Center, Leiden, the Netherlands; 3 The Ritchie Centre, Hudson Institute for Medical Research, Monash University, Melbourne, Victory Australia; 4 Division of Paediatric Intensive Care, Department of Neonatal and Paediatric Intensive Care, Erasmus MC University Medical Center, Rotterdam, the Netherlands; 5 Department of Neonatology, University Medical Center Mannheim, Mannheim, Germany; 6 Division of Neonatology, Department of Paediatrics, Radboudumc University Medical Center, Radboud Institute for Health Sciences, Amalia Children’s Hospital, Nijmegen, The Netherlands; 7 Department of Neonatology, University Hospitals Leuven, Leuven, Belgium; 8 Division of Neonatology, Department of Paediatrics and Adolescent Medicine, Medical University of Graz, Graz, Austria; 9 Department of Paediatrics, Monash University, Melbourne, Victoria Australia; 10 Department of Neonatology and Paediatric Intensive Care Medicine, University of Bonn Children’s Hospital, Bonn, Germany; 11 Department of Medical and Surgical Neonatology, Bambino Gesu Children’s Hospital, IRCCS, Rome, Italy; 12 Department of Paediatric Surgery, Karolinska University Hospital, Department of Women’s and Children’s Health, Karolinska Institutet, Stockholm, Sweden; 13 Department of Epidemiology, Erasmus MC University Medical Center, Rotterdam, the Netherlands; 14 Institute of Health Policy, Management and Evaluation, University of Toronto, Toronto, Canada; 15 Toronto Centre for Liver Disease, University Health Network, Toronto, Canada; 16 Department of Obstetrics and Gynaecology, Erasmus MC University Medical Center, Rotterdam, the Netherlands

**Keywords:** Congenital diaphragmatic hernia, Pulmonary hypertension

## Abstract

**Background:**

Infants born with congenital diaphragmatic hernia (CDH) are at high risk of respiratory insufficiency and pulmonary hypertension. Routine practice includes immediate clamping of the umbilical cord and endotracheal intubation. Experimental animal studies suggest that clamping the umbilical cord guided by physiological changes and after the lungs have been aerated, named physiological-based cord clamping (PBCC), could enhance the fetal-to-neonatal transition in CDH. We describe the statistical analysis plan for the clinical trial evaluating the effects of PBCC versus immediate cord clamping on pulmonary hypertension in infants with CDH (PinC trial).

**Design:**

The PinC trial is a multicentre, randomised controlled trial in infants with isolated left-sided CDH, born ≥ 35.0 weeks of gestation. The primary outcome is the incidence of pulmonary hypertension in the first 24 h after birth. Maternal outcomes include estimated maternal blood loss. Neonatal secondary outcomes include mortality before discharge, extracorporeal membrane oxygenation therapy, and number of days of mechanical ventilation. Infants are 1:1 randomised to either PBCC or immediate cord clamping using variable random permutated block sizes (4–8), stratified by treatment centre and estimated severity of pulmonary hypoplasia (i.e. mild/moderate/severe). At least 140 infants are needed to detect a relative reduction in pulmonary hypertension by one third, with 80% power and 0.05 significance level. A chi-square test will be used to evaluate the hypothesis that PBCC decreases the occurrence of pulmonary hypertension. This plan is written and submitted without knowledge of the collected data. The trial has been ethically approved.

**Trial registration:**

ClinicalTrials.gov NCT04373902 (registered April 2020).

**Supplementary Information:**

The online version contains supplementary material available at 10.1186/s13063-024-08027-7.

## Background

Two thirds of all infants born with congenital diaphragmatic hernia (CDH) develop severe pulmonary hypertension after birth, which is associated with a high risk of mortality [1–5]. Postnatal treatment of pulmonary hypertension mainly consists of pulmonary vasodilators and haemodynamic support including extracorporeal membrane oxygenation (ECMO) therapy. However, the effects of currently used pulmonary vasodilators such as inhaled nitric oxide and intravenous sildenafil in infants with CDH-related pulmonary hypertension are often variable and insufficient [6, 7]. Preclinical studies in an ovine model of diaphragmatic hernia have suggested that the current standard of care including immediate cord clamping contributes to the high pulmonary vascular resistance after birth [8]. Clamping the umbilical cord after the lungs have been aerated potentially attenuates the high pulmonary pressures after birth [8]. To evaluate the effects of this approach, called physiological-based cord clamping (PBCC), on pulmonary hypertension, we are currently conducting a multicentre, randomised controlled trial: PBCC versus immediate cord clamping in infants born with CDH (PinC). The study protocol was published previously [9]. This paper describes the statistical analysis plan (SAP) for the PinC trial in detail and is written and submitted without knowledge of the data.

### Objectives

The primary aim of the PinC trial is to investigate the hypothesis that implementation of PBCC in the stabilisation period of infants born with CDH is effective in reducing the incidence of pulmonary hypertension in the first 24 h after birth when compared to the standard of care of immediate cord clamping.

## Methods/design

### Design and setting

The PinC trial is an international, multicentre, randomised controlled superiority trial in infants born with CDH. Patients are currently recruited from nine tertiary care centres in the Netherlands, Belgium, Austria, Australia, Sweden, Italy, and Germany.

#### Study protocol development and conduct

This trial has been approved by the Medical Ethical Committee of the Erasmus MC University Medical Center, Rotterdam, The Netherlands (MEC2019-0414). The trial is registered with the registry sponsored by the United States National Library of Medicine Clinicaltrials.gov NCT04373902 (registered April 2020). Local ethical approval was obtained from the ethical committees of participating centres before trial initiation. The study is conducted according to the principles of the Declaration of Helsinki, good clinical practice and international rules and regulations on personal data protection [10–12]. Changes in the trial protocol made after initiation of the trial are further explained in this paper.

#### Randomisation and data collection

Inclusion criteria and exclusion criteria are reported in detail in the study protocol [9]. Infants are eligible if diagnosed with an isolated left-sided CDH on prenatal ultrasound with gestational age at delivery ≥ 35.0 weeks [9]. Exclusion criteria are right-sided and bilateral CDH, antenatal diagnosed major associated structural or genetic abnormalities, high urgency caesarean section (intended interval to delivery < 15 min), cases that have been treated during pregnancy with experimental drug therapy aiming to decrease the occurrence of pulmonary hypertension, twin pregnancies in which the infant diagnosed with CDH is born first, multiple birth > 2, and placental abnormalities (i.e. anterior placenta praevia, placental abruption). Written informed consent from both parents is required for inclusion. Eligible fetuses will be randomised 1:1 to either PBCC or the standard approach of immediate cord clamping. Study procedures regarding PBCC and immediate cord clamping are explained in the study protocol [9]. Blinding of the allocation arm during intervention is not possible due to the nature of the intervention. Allocation will be stratified by predicted lung size (determined by observed to expected lung-to-head ratio and liver position, graded as mild/moderate/severe lung hypoplasia, measured between 20 and 26 weeks of gestation or at initial visit) and by treatment centre, using variable random permutated block sizes (4-8) [13]. Randomisation and data collection are performed in the electronic data capture system Castor EDC. This electronic database facilitates on-site data entry and ensures concealment of allocation. Security is guaranteed with login names, login codes, and two factor authentication. Only dedicated and trained co-investigators in each centre receive credentials for Castor EDC and are thus allowed to enter data and randomise patients. Patient data are collected until the end of the study period, which is defined as discharge from the tertiary care hospital or death before discharge depending on which occurs first and with a maximum study duration of 6 months.

Data collection and management are implemented according to good clinical practice guidelines. To ensure feasibility of the trial, participating centres use their local CE-approved devices to assess the outcome measures strictly defined in the trial protocol. Those devices include amongst others resuscitation trolleys, respiratory function monitors, pulse oximeters, echocardiography machines, and laboratory equipment. All participating centres are certified tertiary academic hospitals that can carry out high-standard neonatal intensive care and all the trial-related investigational procedures. To increase reliability of the data, it is promoted to measure echocardiographic parameters in triplicate and averaged, although this is not possible in each centre. Biochemical and haematological outcomes, such as haemoglobin and bilirubin, are assessed in the local certified laboratories. Measurement units will be standardised as has been specified in Tables 1, 2, 3, and 4.

**Table 1:** Baseline characteristics

	**PBCC (*n* = 70)**	**ICC (*n* = 70)**
**Maternal baseline characteristics**		
Age at giving birth (years)	Mean ± SD or median [IQR]	Mean ± SD or median [IQR]
Parity (*n*)	Mean ± SD or median [IQR]	Mean ± SD or median [IQR]
Smoking during pregnancy	*n* (%)	*n* (%)
Preterm premature rupture of the membranes	*n* (%)	*n* (%)
- Gestational age at occurrence (weeks)	Mean ± SD or median [IQR]	Mean ± SD or median [IQR]
Any administration of antenatal corticosteroids		
Mode of delivery:		
- Vaginal birth	*n* (%)	*n* (%)
- Caesarean section	*n* (%)	*n* (%)
◦ Indication:		
▪ Elective	*n* (%)	*n* (%)
▪ Emergency	*n* (%)	*n* (%)
• Reason:		
◦ Fetal distress	*n* (%)	*n* (%)
◦ Failure to progress	*n* (%)	*n* (%)
◦ Maternal indication	*n* (%)	*n* (%)
• Use of general anaesthetics	*n* (%)	*n* (%)
**Fetal baseline characteristics**		
Estimated severity of lung hypoplasia:		
- Mild	*n* (%)	*n* (%)
- Moderate	*n* (%)	*n* (%)
- Severe	*n* (%)	*n* (%)
Liver position:		
- Intra-abdominal	*n* (%)	*n* (%)
- Intrathoracic	*n* (%)	*n* (%)
Observed to expected lung-to-head ratio (%)	Mean ± SD or median [IQR]	Mean ± SD or median [IQR]
- Gestational age at measurement (weeks)	Mean ± SD or median [IQR]	Mean ± SD or median [IQR]
Fetal lung volume right lung (ml)	Mean ± SD or median [IQR]	Mean ± SD or median [IQR]
Fetal lung volume left lung (ml)	Mean ± SD or median [IQR]	Mean ± SD or median [IQR]
Observed to expected fetal lung volume (%)	Mean ± SD or median [IQR]	Mean ± SD or median [IQR]
- Gestational age at measurement (weeks)	Mean ± SD or median [IQR]	Mean ± SD or median [IQR]
Fetoscopic endoluminal tracheal occlusion therapy	*n* (%)	*n* (%)
- Duration of tracheal occlusion (days)	Mean ± SD or median [IQR]	Mean ± SD or median [IQR]
**Neonatal baseline characteristics**		
Sex:		
- Male	*n* (%)	*n* (%)
- Female	*n* (%)	*n* (%)
Birth weight (g)	Mean ± SD or median [IQR]	Mean ± SD or median [IQR]
Gestational age at birth (weeks)	Mean ± SD or median [IQR]	Mean ± SD or median [IQR]
Apgar score at 1 min	Mean ± SD or median [IQR]	Mean ± SD or median [IQR]
Apgar score at 5 min	Mean ± SD or median [IQR]	Mean ± SD or median [IQR]
Umbilical artery pH	Mean ± SD or median [IQR]	Mean ± SD or median [IQR]
Intubation in the delivery room		
- Conventional mechanical ventilation	*n* (%)	*n* (%)
- High frequency oscillatory ventilation	*n* (%)	*n* (%)
◦ Mean airway pressure (cmH_2_O)	Mean ± SD or median [IQR]	Mean ± SD or median [IQR]
◦ Frequency (Hz)	Mean ± SD or median [IQR]	Mean ± SD or median [IQR]
◦ Amplitude (cmH_2_O)	Mean ± SD or median [IQR]	Mean ± SD or median [IQR]
Continuous positive airway pressure in the delivery room	*n* (%)	*n* (%)
High flow in the delivery room	*n* (%)	*n* (%)
Initial fraction of inspired oxygen (%)	Mean ± SD or median [IQR]	Mean ± SD or median [IQR]
Maximum fraction of inspired oxygen (%)	Mean ± SD or median [IQR]	Mean ± SD or median [IQR]
Maximum positive end-expiratory pressure (cmH_2_O)	Mean ± SD or median [IQR]	Mean ± SD or median [IQR]
Maximum peak inspiratory pressure (cmH_2_O)	Mean ± SD or median [IQR]	Mean ± SD or median [IQR]
Maximum flow (l/min)	Mean ± SD or median [IQR]	Mean ± SD or median [IQR]
Body temperature at admission to the intensive care unit (degrees Celsius)	Mean ± SD or median [IQR]	Mean ± SD or median [IQR]
Haemoglobin level at admission to the intensive care unit (mmol/l)	Mean ± SD or median [IQR]	Mean ± SD or median [IQR]

*ICC*,Immediate cord clamping, *IQR* Interquartile range, *PBCC* Physiological-based cord clamping

**Table 2:** Primary and secondary outcomes

	**PBCC (*n* = 70)**	**ICC (*n* = 70)**	***p* value**	**Difference**^a^	**Relative risk**^b^
**Primary outcome**					
Pulmonary hypertension (defined as at least 2 out of 4 criteria for pulmonary hypertension *or* need for ECMO therapy in the first 24 h) after birth *in the total study population*	*n* (%)	*n* (%)	*p*	Difference (95% CI)	RR (95% CI)
Pulmonary hypertension or need for ECMO therapy in the first 24 h after birth* in subgroups with estimated:*					
*- Mild pulmonary hypoplasia*	*n* (%)	*n* (%)	*p*	Difference (95% CI)	RR (95% CI)
*- Moderate pulmonary hypoplasia*	*n* (%)	*n* (%)	*p*	Difference (95% CI)	RR (95% CI)
*- Severe pulmonary hypoplasia*	*n* (%)	*n* (%)	*p*	Difference (95% CI)	RR (95% CI)
**Secondary outcomes**^c^					
Mortality before discharge from tertiary care hospital	*n* (%)	*n* (%)		Difference (95% CI)	RR (95% CI)
Presence of ≥ 3 criteria for pulmonary hypertension or extracorporeal membrane oxygenation within 24 h after birth	*n* (%)	*n* (%)		Difference (95% CI)	RR (95% CI)
ECMO therapy	*n* (%)	*n* (%)		Difference (95% CI)	RR (95% CI)
Number of days needing supplemental oxygen	Mean ± SD and median [IQR]	Mean ± SD and median [IQR]		Difference (95% CI)	
Number of days on mechanical ventilation	Mean ± SD and median [IQR]	Mean ± SD and median [IQR]		Difference (95% CI)	
Number of days of admission to tertiary care hospital	Mean ± SD and median [IQR]	Mean ± SD and median [IQR]		Difference (95% I)	
Postpartum haemorrhage (estimated maternal blood loss > 1000 ml)	*n* (%)	*n* (%)		Difference (95% CI)	RR (95% CI)

*CI* Confidence interval, *ECMO* Extracorporeal membrane oxygenation, *ICC* Immediate cord clamping, *IQR* Interquartile range, *OR* Odds ratio, *PBCC* Physiological-based cord clamping, *RR* Relative risk, *SD* Standard deviation

^a^Differences will be calculated as the absolute difference in percentages for dichotomous data or as the difference in medians and means for continuous data

^b^Relative risks will only be calculated for dichotomous data

^c^Secondary outcomes will be reported in the total study population and in the subgroup with survivors, respectively

**Table 3:** Exploratory outcomes

	**PBCC (*n* = 70)**	**ICC (*n* = 70)**
**Treatment related exploratory outcomes**		
Protocol violations (yes/no)	*n* (%)	*n* (%)
Time interval between birth and positioning of the baby on the resuscitation table (s)	Mean ± SD or median [IQR]	Mean ± SD or median [IQR]
Time interval between birth and start of respiratory support (s)	Mean ± SD or median [IQR]	Mean ± SD or median [IQR]
Time interval between birth and time to ‘stable’ (heart rate > 100 bpm, saturation > 85%, and FiO_2_ < 0.5; s)	Mean ± SD or median [IQR]	Mean ± SD or median [IQR]
Time interval between birth and cord clamping (s)	Mean ± SD or median [IQR]	Mean ± SD or median [IQR]
**Maternal exploratory outcomes**		
Estimated maternal blood loss during delivery (ml)	Mean ± SD or median [IQR]	Mean ± SD or median [IQR]
Need for blood transfusion during admission (yes/no)	*n* (%)	*n* (%)
Surgical site infection after caesarean section (yes/no)	*n* (%)	*n* (%)
Other maternal morbidity (yes/no)	*n* (%)	*n* (%)
**Neonatal exploratory outcomes**		
Highest oxygenation index in the first 24 h	Mean ± SD or median [IQR]	Mean ± SD or median [IQR]
Highest pre-postductal SpO_2_ difference in the first 24 h (%)	Mean ± SD or median [IQR]	Mean ± SD or median [IQR]
Number of criteria for pulmonary hypertension present	Mean ± SD or median [IQR]	Mean ± SD or median [IQR]
Systemic blood pressure during echocardiography (mmHg)	Mean ± SD or median [IQR]	Mean ± SD or median [IQR]
ECMO duration (days)	Mean ± SD or median [IQR]	Mean ± SD or median [IQR]
ECMO complications (yes/no)	*n* (%)	*n* (%)
Pulmonary hypertension requiring therapy at:		
- Day 7 (yes/no)	*n* (%)	*n* (%)
- Day 14 (yes/no)	*n* (%)	*n* (%)
- Day 21 (yes/no)	*n* (%)	*n* (%)
- Day 28 (yes/no)	*n* (%)	*n* (%)
- Discharge (yes/no)	*n* (%)	*n* (%)
Use of pulmonary vasodilators:		
- Sildenafil (yes/no)	*n* (%)	*n* (%)
◦ Number of days	Mean ± SD or median [IQR]	Mean ± SD or median [IQR]
- Prostaglandin E (yes/no)	*n* (%)	*n* (%)
◦ Number of days	Mean ± SD or median [IQR]	Mean ± SD or median [IQR]
- Bosentan (yes/no)	*n* (%)	*n* (%)
◦ Number of days	Mean ± SD or median [IQR]	Mean ± SD or median [IQR]
- Prostacyclins (yes/no)	*n* (%)	*n* (%)
◦ Number of days	Mean ± SD or median [IQR]	Mean ± SD or median [IQR]
- Milr◦ inone (yes/no)	*n* (%)	*n* (%)
◦ Number of days	Mean ± SD or median [IQR]	Mean ± SD or median [IQR]
- iNO (yes/no)	*n* (%)	*n* (%)
◦ Number of days	Mean ± SD or median [IQR]	Mean ± SD or median [IQR]
◦ Response to iNO (yes/no)	*n* (%)	*n* (%)
◦ iNO started as routine management (yes/no)	*n* (%)	*n* (%)
Use of inotropes in the first 72 h:		
- Adrenalin (yes/no)	*n* (%)	*n* (%)
◦ Maximum dosage (mcg/kg/min)	Mean ± SD or median [IQR]	Mean ± SD or median [IQR]
- Dobutamine (yes/no)	*n* (%)	*n* (%)
◦ Maximum dosage (mcg/kg/min)	Mean ± SD or median [IQR]	Mean ± SD or median [IQR]
- Dopamine (yes/no)	*n* (%)	*n* (%)
◦ Maximum dosage (mcg/kg/min)	Mean ± SD or median [IQR]	Mean ± SD or median [IQR]
- Milrinone (yes/no)	*n* (%)	*n* (%)
◦ Maximum dosage (mcg/kg/min)	Mean ± SD or median [IQR]	Mean ± SD or median [IQR]
- Noradrenalin (yes/no)	*n* (%)	*n* (%)
◦ Maximum dosage (mcg/kg/min)	Mean ± SD or median [IQR]	Mean ± SD or median [IQR]
- Vasopressin (yes/no)	*n* (%)	*n* (%)
◦ Maximum dosage (units/kg/min)	Mean ± SD or median [IQR]	Mean ± SD or median [IQR]
Administration of a bolus of fluid in the first 24 h (yes/no)	*n* (%)	*n* (%)
Frequency of bolus of fluid in first 24 h	Mean ± SD or median [IQR]	Mean ± SD or median [IQR]
Total volume of fluid therapy in the first 24 h (ml/kg)	Mean ± SD or median [IQR]	Mean ± SD or median [IQR]
Culture proven early onset sepsis < 72 h (yes/no)	*n* (%)	*n* (%)
- Start day	Mean ± SD or median [IQR]	Mean ± SD or median [IQR]
Culture proven late onset sepsis > 72 h (yes/no)	*n* (%)	*n* (%)
- Start day	Mean ± SD or median [IQR]	Mean ± SD or median [IQR]
Hyperbilirubinemia requiring therapy (yes/no)	*n* (%)	*n* (%)
- Maximum bilirubin level (mg/dl)	Mean ± SD or median [IQR]	Mean ± SD or median [IQR]
Phototherapy (yes/no)	*n* (%)	*n* (%)
- Duration of phototherapy (days)	Mean ± SD or median [IQR]	Mean ± SD or median [IQR]
Exchange transfusion (yes/no)	*n* (%)	*n* (%)
Cerebral complications:		
- Haemorrhage (yes/no)	*n* (%)	*n* (%)
- Infarction (yes/no)	*n* (%)	*n* (%)
- Other (yes/no)	*n* (%)	*n* (%)
Surgical repair diaphragm (yes/no)	*n* (%)	*n* (%)
Day of surgery	Mean ± SD or median [IQR]	Mean ± SD or median [IQR]
Use of patch during surgery (yes/no)	*n* (%)	*n* (%)
Surgical approach:		
- Laparotomic (yes/no)	*n* (%)	*n* (%)
- Thoracoscopic (yes/no)	*n* (%)	*n* (%)
- Laparoscopic (yes/no)	*n* (%)	*n* (%)
Diaphragmatic defect size [18]:		
- A	*n* (%)	*n* (%)
- B	*n* (%)	*n* (%)
- C	*n* (%)	*n* (%)
- D	*n* (%)	*n* (%)
- Unknown	*n* (%)	*n* (%)
Number of days on:		
- NIPPV/NIV (days)	Mean ± SD or median [IQR]	Mean ± SD or median [IQR]
- CPAP/high flow > 2 L (days)	Mean ± SD or median [IQR]	Mean ± SD or median [IQR]
- Low flow ≤ 2 L (days)	Mean ± SD or median [IQR]	Mean ± SD or median [IQR]
Oxygen dependency on day 28 (yes/no)	*n* (%)	*n* (%)
Participation in other randomised controlled trials (yes/no)	*n* (%)	*n* (%)
Number of days on intensive care unit	Mean ± SD or median [IQR]	Mean ± SD or median [IQR]
Discharge without oxygen dependency (yes/no)	*n* (%)	n (%)
Discharged on pulmonary vasodilators (yes/no)	*n* (%)	*n* (%)
Discharged with palliative care (yes/no)	*n* (%)	*n* (%)
Number of days alive if death before discharge	Mean ± SD or median [IQR]	Mean ± SD or median [IQR]

*CPAP* Continuous positive airway pressure, *ECMO* Extracorporeal membrane oxygenation, *ICC* Immediate cord clamping, *iNO*, Inhaled nitric oxide, *IQR* Interquartile range, *NIPPV* Non-invasive positive pressure ventilation; non-invasive ventilation, *PBCC* Physiological-based cord clamping, *SpO*_*2*_ Oxygen saturation

**Table 4:** Echocardiographic parameters [15]

	**PBCC (*n* = 70)**	**ICC (*n* = 70)**
Right ventricular systolic pressure (mmHg)	Mean ± SD or median [IQR]	Median [IQR]
Right ventricular size:		
- Normal (yes/no)	*n* (%)	*n* (%)
- Dilated (yes/no)	*n* (%)	*n* (%)
Right ventricular function:		
- Normal (yes/no)	*n* (%)	*n* (%)
- Impaired (yes/no)	*n* (%)	*n* (%)
Pulmonary artery acceleration time (PAAT; ms)	Mean ± SD or median [IQR]	Median [IQR]
Right ventricular ejection time (RVET; ms)	Mean ± SD or median [IQR]	Median [IQR]
PAAT to RVET ratio	Mean ± SD or median [IQR]	Median [IQR]
Interventricular septum configuration:		
- Normal (O-shaped; yes/no)	*n* (%)	*n* (%)
- Flattened (D-shaped; yes/no)	*n* (%)	*n* (%)
- Displaced (crescent-shaped; yes/no)	*n* (%)	*n* (%)
Left ventricular dimension parallel to the septum (D1)	Mean ± SD or median [IQR]	Median [IQR]
Left ventricular dimension perpendicular to the septum (D2)	Mean ± SD or median [IQR]	Median [IQR]
Left ventricular systolic eccentricity index: D1/D2	Mean ± SD or median [IQR]	Median [IQR]
Tricuspid regurgitation (yes/no)	*n* (%)	*n* (%)
Peak velocity of tricuspid regurgitation (m/s)	Mean ± SD or median [IQR]	Median [IQR]
Tricuspid annular plane systolic excursion (mm)	Mean ± SD or median [IQR]	Median [IQR]
Systolic duration (SD; ms)	Mean ± SD or median [IQR]	Median [IQR]
Diastolic duration (DD; ms)	Mean ± SD or median [IQR]	Median [IQR]
Right ventricular SD/DD ratio	Mean ± SD or median [IQR]	Median [IQR]
Transductal shunting direction:		
- Right-to-left (yes/no)	*n* (%)	*n* (%)
- Left-to-right (yes/no)	*n* (%)	*n* (%)
- Bidirectional (yes/no)	*n* (%)	*n* (%)
Transductal shunting peak flow velocity (m/s)	Mean ± SD or median [IQR]	Median [IQR]
Interatrial shunting direction		
- Right-to-left (yes/no)	*n* (%)	*n* (%)
- Left-to-right (yes/no)	*n* (%)	*n* (%)
- Bidirectional (yes/no)	*n* (%)	*n* (%)

*ICC* Immediate cord clamping, *IQR*, interquartile range, *PBCC*, physiological-based cord clamping

#### Baseline characteristics

Baseline characteristics will be collected for mothers and infants and will be presented in the final report of the trial. All collected data are depicted in Table 1.

#### Primary outcome

The primary outcome is pulmonary hypertension diagnosed in the first 24 h after birth based on a combination of clinical and echocardiographic criteria, as was described in the study protocol [9]. Clinical parameters are as follows: (1) a difference between preductal and postductal oxygen saturation > 10% for a minimum of 15 min, with the specification of 15 min being added to the protocol after trial commencement because a single measurement of > 10% is likely due to a measurement error; (2) oxygenation index > 20. Echocardiographic parameters are as follows: (1) right ventricular systolic pressure ≥ 2/3 systemic systolic pressure; (2) right ventricle dilatation/septal displacement or right ventricular dysfunction ± left ventricular dysfunction [14, 15]. Pulmonary hypertension is present if at least 2 out of 4 criteria are present *or* if the infant requires ECMO therapy within the first 24 h after birth [14].

The initial version of the research protocol described echocardiographic evaluation between 12 to 24 h after birth. To guarantee feasibility in all centres, the trial team changed the evaluation period to within the first 24 h after birth, as routine evaluation in some centres takes places within the first 12 h. This change was reported to and approved by the medical ethical committee of the Erasmus MC in March 2020, before inclusion of the first patient. Furthermore, aiming to limit bias, the primary outcome was refined with the statement about ECMO therapy after discussions with additional centres in April 2021.

#### Secondary outcomes and exploratory outcomes

To limit type I errors, we predefined a limited number of secondary outcomes that will be included in formal statistical analyses. The choice of secondary outcomes was based on clinical relevance and existing literature and includes the following: (1) mortality before discharge from the tertiary care hospital, (2) presence of ≥ 3 criteria for pulmonary hypertension or extracorporeal membrane oxygenation within 24 h after birth, (3) ECMO therapy, (4) duration of supplemental oxygen need, (5) duration of mechanical ventilation, (6) duration of admission to the tertiary care hospital, and the safety parameter (7) postpartum haemorrhage (Table 2). All other secondary outcomes will be considered exploratory outcomes that will not be included in formal statistical testing. Additional to the exploratory outcomes depicted in Table 3, we will collect the following data: echocardiographic confirmation of the presence of pulmonary hypertension requiring therapy on days 7, 14, 21, and 28 and at discharge; the response to iNO defined as one of the following criteria: a decline of 10–20% in pre-postductal SpO_2_ difference, or an increase of 10–20% in PaO_2_, or improvement in haemodynamic parameters meaning 10% increase in mean blood pressure, or a decrease in lactate levels [6]. The number of days needing supplemental oxygen is defined as each calendar day on which the infant required FiO_2_ > 21% for any duration that day. For each calendar day of respiratory support, only the modality with the highest level of support applied on that day will be counted. Causes of death for deceased infants will be summarised in the final report. To objectify the echocardiographic criteria in the primary outcome, we will collect specific echocardiographic parameters in the first 24 h after birth, as depicted in Table 4 [15]. Where possible, the investigator evaluating the echocardiography is blinded to allocation, which will not be feasible in all centres due to limited human resources in routine practice. The first echocardiography including the parameters mentioned in Table 4 will be analysed.

Finally, continuous physiological measurements will be collected in the first 72 h if feasible, including the following: heart rate (bpm), preductal and postductal saturation (%), cerebral oxygenation (%), mean arterial blood pressure (mmHg), arterial partial pressure of oxygen (PaO_2_, kPa), and respiratory support settings (mean airway pressure in cmH_2_O, fraction of inspired oxygen in %, flow in L/min). Limited to infants born in the Erasmus MC, parental perception and appreciation of the approach during birth and stabilisation of their infant will be evaluated with a short questionnaire in both treatment arms. This questionnaire includes rating of 7 items on a 5-point scale and an open question. Topics include parental anxiety, safety, size of the team present, and provision of information. The results from physiological measurements and the parental questionnaire will not be analysed in the main report of the study but will be explored and reported separately.

#### Safety

As CDH is a condition already associated with a significant risk of complications, serious morbidity is often inherent to the disease and unrelated to the intervention that is under evaluation in this trial. Therefore, we have specified context-specific SAEs that are reported to the data and safety monitoring board (DSMB) and METC on an annual base and that are collected as secondary outcomes: oxygen dependency on day 28, sepsis, cerebral complications, and ECMO therapy. Non-context specific SAEs are reported to the METC within 15 days after the sponsor has first knowledge of the SAE. Postpartum haemorrhage (estimated maternal blood loss > 1000 ml) is considered a safety parameter, and the sponsor will as such report this serious adverse event (SAE) to the METC within 7 days of first knowledge.

A DSMB was established to advise the principal investigator in protecting trial safety. Members of this committee are two neonatologists, an obstetrician, and a statistician. As stated in the protocol, the DSMB will conduct two interim statistical analyses on safety during the course of this study, after approximately 25% and 50% of the total required patients have completed their primary outcome. Outcomes included in the interim analysis on safety include the abovementioned context-specific SAEs, neonatal mortality, and postpartum haemorrhage. The first interim analysis has been conducted and resulted in the DSMB advising in favour of continuing the trial.

### Statistical methods specified in the study protocol

#### Sample size calculation

As has been reported in the study protocol, the background incidence of pulmonary hypertension was previously reported at 69.7% in the first week after birth [9, 16]. Based on a suggested clinically relevant reduction in pulmonary hypertension incidence of one third, a total sample size of at least 140 infants was calculated with 80% power and a two-sided type I error of 5%.

#### Originally proposed analyses

After start of the trial, the initially proposed analysis plan was updated to increase feasibility of the study in all participating centres and to limit bias where possible. Here, we present our updated statistical analysis plan.

#### Interim analyses and safety reporting

As was specified in the study protocol, no interim analyses on efficacy will be performed. Only two interim analyses on safety are planned, after 25% and 50% of the total required patients have completed the primary outcome. The only stopping condition will be concerns regarding safety outcomes. The decision to terminate or continue the trial is advised by the DSMB. The interim safety analyses include SAEs and the pre-specified context-specific safety outcomes listed as exploratory outcomes. Before each interim analysis, the DSMB will receive a report that includes blinded data on safety outcomes. On request of the DSMB, treatment allocation can be unblinded. The first safety interim analysis, after 25% of the total required patients had completed the primary outcome, was performed in 2022. The DSMB advised to continue the trial based on this interim analysis. In the final report of the trial, a list of SAEs and reasons of mortality will be reported by allocation arm in supplementary tables. SAEs and context-specific safety outcomes will be reported as is described in the ‘Statistical analyses’ section on secondary outcomes.

### Statistical analysis plan

#### Overall principle

Statistical significance is set at *p* < 0.05, using two-sided tests. For all relevant outcomes, a 95% confidence interval (CI) will be reported. Statistical analyses will be performed using the computing environment R (R Core Team (2020), Vienna, Austria).

#### Data handling

Potential outliers will be investigated, and extreme outliers, defined as being more than three times the interquartile (IQR) range below the first quartile or above the third quartile, will be listed individually in a supplement to the main analysis. If it can be reasonably assumed that those extreme outliers are due to an error in the data, they will be excluded from all analyses. The data set will only be locked after completion of the data set, data cleaning, and data validation. The statistical analyses for significant differences are done on a blinded data set and will be carried out after locking the data set, which can only be reversed in case of exceptional circumstances and after agreement of the trial team that consists of the principal investigator, data managers, and the trial statistician.

#### Definition of analysis sets

The primary outcome will be analysed in a modified intention to-treat population to estimate the realised benefit of the intention to do PBCC over immediate cord clamping in patients with a prenatal diagnosis of CDH. The modified intention-to-treat population includes all patients that are randomised to a particular treatment arm (PBCC or immediate cord clamping), independent of actual treatment received, protocol deviations, or post-randomisation ineligibility. However, two exclusions apply: (1) patients without a confirmed prenatal diagnosis of CDH will be excluded from all analyses; (2) patients whose parents withdraw consent will be excluded from all analyses.

### Statistical analyses

#### Patient flow

**Fig. 1:**
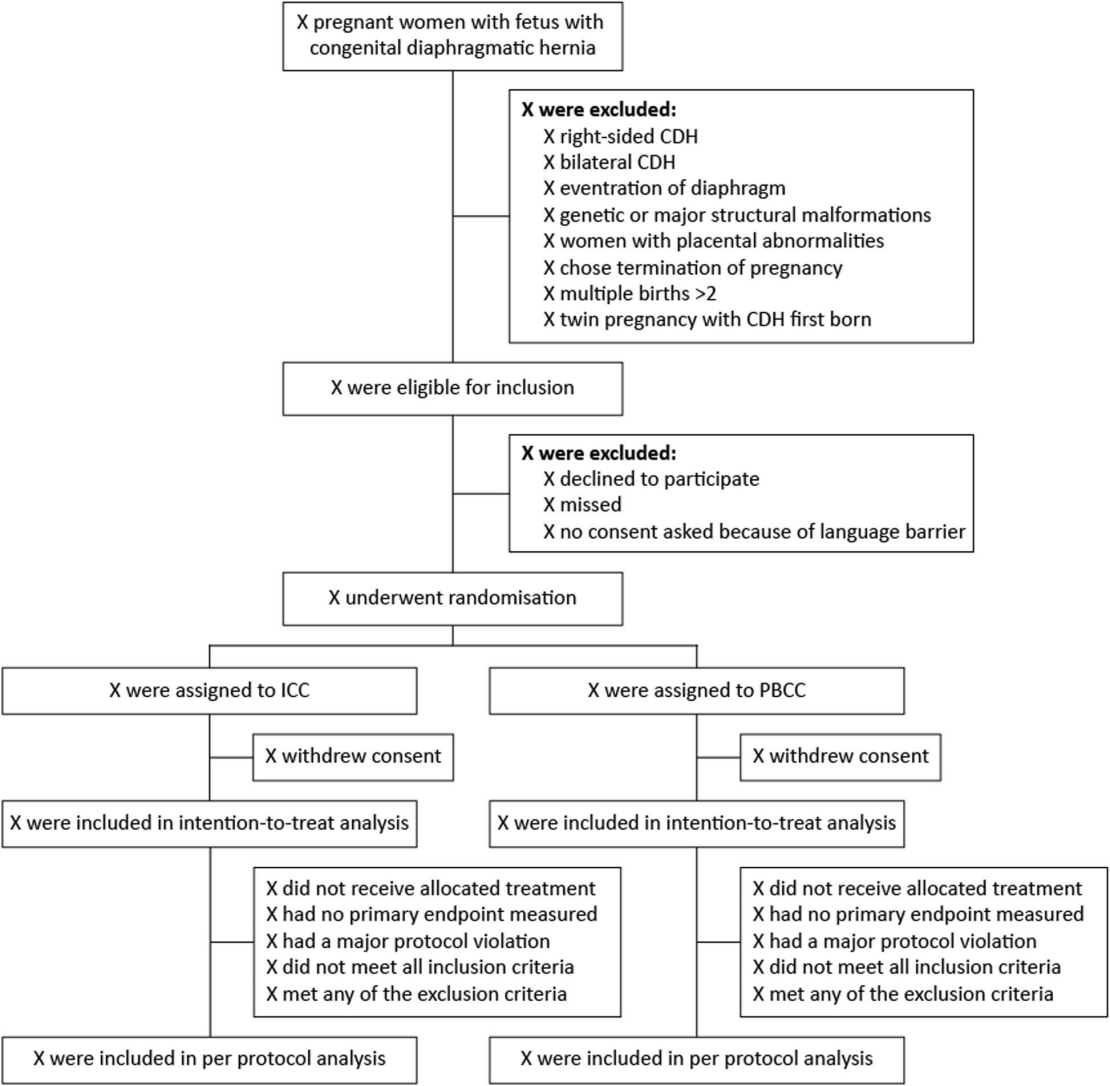
Flowchart of inclusion. CDH, congenital diaphragmatic hernia; ICC, immediate cord clamping; PBCC, physiological-based cord clamping

Figure 1 shows the expected patient flow. A similar figure, completed with numbers per category, will be included in the final report of the trial. We will summarise the reasons why patients are not eligible and the reasons for not including eligible patients if reported. Protocol deviations, defined as deviations in eligibility criteria and patients not being stabilised according to the allocated protocol as set forth in the study protocol, will also be reported.

### Baseline characteristics

All baseline characteristics will be described for each allocation arm of the trial as depicted in Table 1. No formal statistical testing will be performed on baseline characteristics. Continuous data will be reported as mean ± SD or median [IQR] depending on distribution, and categorical data will be reported as counts and percentages.

#### Primary outcome

The effect of PBCC on the primary outcome will be analysed as a complete case analysis in the intention-to-treat population. The main effect of PBCC on pulmonary hypertension will be analysed using the chi-square test. To explore the effect of PBCC on pulmonary hypertension per strata, subgroup analysis per strata (estimated mild/moderate/severe pulmonary hypoplasia) will be performed. An additional sensitivity analysis applying mixed effect models will be conducted to investigate the specific strata. The primary outcome will also be reported as absolute differences in percentages with 95% CI and relative risks with 95% CI to compare the intervention group with the control group (Table 2).

Analysis of the primary outcome will be based on complete cases and by protocol the independent variables in this multivariable analysis cannot be missing as these are required for randomisation. In the rare event that evaluation of the primary outcome has not been performed in the first 24 h, e.g. in the event of mortality before echocardiographic evaluation, the dependent outcome (i.e. pulmonary hypertension) will be missing. To evaluate the robustness of our findings and potential bias by such missing data, we will perform a sensitivity analysis. In this sensitivity analysis, missing values will be imputed by using the ‘worst case’ observed in cases in which the primary outcome was assessed.

#### Secondary outcomes

To limit multiplicity, formal statistical analyses will be carried out for only a limited number of pre-defined secondary outcomes that were regarded most relevant from a clinical perspective (Table 2). When mortality competes with outcomes, the risk for these outcomes can be underestimated. Therefore, these secondary outcomes will be reported in both the group of survivors and the total study population. For continuous variables, we will report absolute differences in means and medians with 95% CI. For dichotomous variables, we will report relative risks with 95% CI and absolute differences in percentages with 95% CI. The results will not be adjusted for multiplicity and *p*-values will not be calculated. Additionally, the distribution of the data for each of the pre-defined continuous secondary outcomes will be presented in histograms.

All possible effort will be made to complete the dataset and we expect that data on the predefined secondary outcomes will be present in nearly all infants. Hence, we will not use imputation to complete the dataset in case of missing values. The number of missing data in secondary outcomes will be reported.

#### Exploratory secondary outcomes

Descriptive statistics will be used to report all exploratory secondary outcomes—including context-specific safety outcomes—in the intervention group and control group, separately, and formal statistical testing will thus not be performed. Continuous data will be reported as mean ± SD or median [IQR] depending on distribution, and categorical data will be reported as counts and percentages.

### Trial reporting and status

The trial will be reported following the principles laid out in the CONSORT statement [17]. The trial was initiated in the Erasmus MC in May 2020. Eight additional centres started recruitment of patients between August 2020 and March 2024. We anticipate on including the final patient end-2024.

## Supplementary Information

**Supplementary Material 1.** 

## Data Availability

Data sharing is not applicable to this article as no datasets were generated or analysed during the current study.

## References

[1] Wynn J, Krishnan U, Aspelund G, Zhang Y, Duong J, Stolar CJ, et al. Outcomes of congenital diaphragmatic hernia in the modern era of management. J Pediatr. 2013;163(1):114-9 e1. 23375362 10.1016/j.jpeds.2012.12.036 PMC3692597

[2] Langham MR Jr, Kays DW, Ledbetter DJ, Frentzen B, Sanford LL, Richards DS. Congenital diaphragmatic hernia. Epidemiology and outcome. Clin Perinatol. 1996;23(4):671–88. 8982563

[3] Levison J, Halliday R, Holland AJ, Walker K, Williams G, Shi E, et al. A population-based study of congenital diaphragmatic hernia outcome in New South Wales and the Australian Capital Territory, Australia, 1992–2001. J Pediatr Surg. 2006;41(6):1049–53. 16769332 10.1016/j.jpedsurg.2006.01.073

[4] Stege G, Fenton A, Jaffray B. Nihilism in the 1990s: the true mortality of congenital diaphragmatic hernia. Pediatrics. 2003;112(3 Pt 1):532–5. 12949279 10.1542/peds.112.3.532

[5] van den Hout L, Schaible T, Cohen-Overbeek TE, Hop W, Siemer J, van de Ven K, et al. Actual outcome in infants with congenital diaphragmatic hernia: the role of a standardized postnatal treatment protocol. Fetal Diagnosis Ther. 2011;29(1):55–63. 10.1159/000322694 21325859

[6] Snoek KG, Reiss IK, Greenough A, Capolupo I, Urlesberger B, Wessel L, et al. Standardized postnatal management of infants with congenital diaphragmatic hernia in Europe: the CDH EURO consortium consensus - 2015 update. Neonatology. 2016;110(1):66–74. 27077664 10.1159/000444210

[7] Noh CY, Chock VY, Bhombal S, Danzer E, Patel N, Dahlen A, et al. Early nitric oxide is not associated with improved outcomes in congenital diaphragmatic hernia. Pediatr Res. 2023. 10.1038/s41390-023-02491-8 36725908

[8] Kashyap AJ, Hodges RJ, Thio M, Rodgers KA, Amberg BJ, McGillick EV, et al. Physiologically based cord clamping improves cardiopulmonary haemodynamics in lambs with a diaphragmatic hernia. Arch Dis Child Fetal Neonatal Ed. 2019. 10.1136/archdischild-2019-316906 31123056

[9] Horn-Oudshoorn EJJ, Knol R, Te Pas AB, Hooper SB, Cochius-den Otter SCM, Wijnen RMH, et al. Physiological-based cord clamping versus immediate cord clamping for infants born with a congenital diaphragmatic hernia (PinC): study protocol for a multicentre, randomised controlled trial. BMJ Open. 2022;12(3):e054808. 35304395 10.1136/bmjopen-2021-054808 PMC8935184

[10] World Medical Association (WMA). WMA Declaration of Helsinki – ethical principles for medical research involving human subjects. Available from: https://www.wma.net/policies-post/wma-declaration-of-helsinki-ethical-principles-for-medicalresearch-involving-human-subjects/. 19886379

[11] Wet medisch-wetenschappelijk onderzoek met mensen. Available from: https://wetten.overheid.nl/BWBR0009408/2022-07-01/0.

[12] Central Committee on Research Involving Human Subjects (CCMO). Standard research file [Available from: https://english.ccmo.nl/investigators/standard-research-file-for-research-subject-to-the-dutch-wmo-act.

[13] Deprest JA, Flemmer AW, Gratacos E, Nicolaides K. Antenatal prediction of lung volume and in-utero treatment by fetal endoscopic tracheal occlusion in severe isolated congenital diaphragmatic hernia. Semin Fetal Neonat Med. 2009;14(1):8–13. 10.1016/j.siny.2008.08.010 18845492

[14] Cochius-den Otter S, Schaible T, Greenough A, van Heijst A, Patel N, Allegaert K, et al. The CoDiNOS trial protocol: an international randomised controlled trial of intravenous sildenafil versus inhaled nitric oxide for the treatment of pulmonary hypertension in neonates with congenital diaphragmatic hernia. BMJ Open. 2019;9(11):e032122. 31694851 10.1136/bmjopen-2019-032122 PMC6858099

[15] de Boode WP, Singh Y, Molnar Z, Schubert U, Savoia M, Sehgal A, et al. Application of neonatologist performed echocardiography in the assessment and management of persistent pulmonary hypertension of the newborn. Pediatr Res. 2018;84(Suppl 1):68–77. 30072805 10.1038/s41390-018-0082-0 PMC6257221

[16] Putnam LR, Tsao K, Morini F, Lally PA, Miller CC, Lally KP, et al. Evaluation of variability in inhaled nitric oxide use and pulmonary hypertension in patients with congenital diaphragmatic hernia. JAMA Pediatr. 2016;170(12):1188–94. 27723858 10.1001/jamapediatrics.2016.2023

[17] Butcher NJ, Monsour A, Mew EJ, Chan AW, Moher D, Mayo-Wilson E, et al. Guidelines for reporting outcomes in trial reports: the CONSORT-Outcomes 2022 extension. Jama. 2022;328(22):2252–64. 36511921 10.1001/jama.2022.21022

[18] Tsao K, Lally KP. The Congenital Diaphragmatic Hernia Study Group: a voluntary international registry. Semin Pediatr Surg. 2008;17(2):90–7. 18395658 10.1053/j.sempedsurg.2008.02.004

