# Correction: Multicentre, randomised controlled trial of physiological-based cord clamping versus immediate cord clamping in infants with a congenital diaphragmatic hernia (PinC): statistical analysis plan

**DOI:** 10.1186/s13063-025-08939-y

**Published:** 2025-07-03

**Authors:** Emily J. J. Horn-Oudshoorn, Marijn J. Vermeulen, Ronny Knol, Rebekka Bout-Rebel, Arjan B. te Pas, Stuart B. Hooper, Suzan C. M. Cochius-den Otter, Rene M. H. Wijnen, Kelly J. Crossley, Neysan Rafat, Thomas Schaible, Willem P. de Boode, Anne Debeer, Berndt Urlesberger, Calum T. Roberts, Florian Kipfmueller, Irma Capolupo, Carmen M. Burgos, Bettina E. Hansen, Irwin K. M. Reiss, Philip L. J. DeKoninck

**Affiliations:** 1 Division of Neonatology, Department of Neonatal and Paediatric Intensive Care, Erasmus MC University Medical Center, Rotterdam, the Netherlands; 2 Division of Neonatology, Department of Paediatrics, Leiden University Medical Center, Leiden, the Netherlands; 3 The Ritchie Centre, Hudson Institute for Medical Research, Monash University, Melbourne, Victory Australia; 4 Division of Paediatric Intensive Care, Department of Neonatal and Paediatric Intensive Care, Erasmus MC University Medical Center, Rotterdam, the Netherlands; 5 Department of Neonatology, University Medical Center Mannheim, Mannheim, Germany; 6 Division of Neonatology, Department of Paediatrics, Radboudumc University Medical Center, Radboud Institute for Health Sciences, Amalia Children’s Hospital, Nijmegen, The Netherlands; 7 Department of Neonatology, University Hospitals Leuven, Louvain, Belgium; 8 Division of Neonatology, Department of Paediatrics and Adolescent Medicine, Medical University of Graz, Graz, Austria; 9 Department of Paediatrics, Monash University, Melbourne, VIC Australia; 10 Department of Neonatology and Paediatric Intensive Care Medicine, University of Bonn Children’s Hospital, Bonn, Germany; 11 Department of Medical and Surgical Neonatology, Bambino Gesu Children’s Hospital, IRCCS, Rome, Italy; 12 Department of Paediatric Surgery, Karolinska University Hospital, Department of Women’s and Children’s Health, Karolinska Institutet, Stockholm, Sweden; 13 Department of Epidemiology, Erasmus MC University Medical Center, Rotterdam, the Netherlands; 14 Institute of Health Policy, Management and Evaluation, University of Toronto, Toronto, Canada; 15 Toronto Centre for Liver Disease, University Health Network, Toronto, Canada; 16 Department of Obstetrics and Gynaecology, Erasmus MC University Medical Center, Rotterdam, the Netherlands

**Correction: Trials 25, 198 (2024)**

**https://doi.org/10.1186/s13063-024–08027−7**

Following the publication of the original article [1], we were notified of a correction in the section titled *Definition of analysis sets.*

**Originally published description:**

“Defnition of analysis sets Te primary outcome will be analysed in the intentionto-treat population to estimate the realised beneft of the intention to do PBCC over immediate cord clamping. Te intention-to-treat population includes all patients that are randomised to a particular treatment arm (PBCC or immediate cord clamping), independent of actual treatment received, protocol deviations, or exclusion criteria. Patients will only be excluded from the study and thus from all analyses if parental consent is withdrawn. A secondary analysis will be performed in the per-protocol population to estimate the beneft of using PBCC— instead of having the intention to—over immediate cord clamping in the target population. Te per-protocol population includes all randomised patients who completed the protocol for the arm they were assigned to, had the primary endpoint measured, had no major protocol violations, met all inclusion criteria, and did not meet any of the exclusion criteria. Relevant major protocol violations are limited to equipment-related decisions to deviate from the assigned protocol. For example, infants that are allocated to PBCC but receive immediate cord clamping due to the resuscitation trolley not being present will be analysed in the PBCC group in the intention-to-treat analysis but will be excluded from the per-protocol populations. Analysing these infants in the immediate cord clamping group could introduce bias, as we assume that cross-over will not be random. Additionally, we do not anticipate that infants receive PBCC despite being randomised for immediate cord clamping.”

**Corrected description:**

“The primary outcome will be analysed in a modified intention to-treat population to estimate the realised benefit of the intention to do PBCC over immediate cord clamping in patients with a prenatal diagnosis of CDH. The modified intention-to-treat population includes all patients that are randomised to a particular treatment arm (PBCC or immediate cord clamping), independent of actual treatment received, protocol deviations, or post-randomisation ineligibility. However, two exclusions apply: (1) patients without a confirmed prenatal diagnosis of CDH will be excluded from all analyses; (2) patients whose parents withdraw consent will be excluded from all analyses.”

By excluding neonates who did not have a prenatal diagnosis of congenital diaphragmatic hernia (CDH), the authors avoid diluting the treatment effect with data from patients who are not part of the target population. Importantly, this does not introduce bias, as the exclusion is based on objective diagnostic confirmation unrelated to treatment allocation or outcome. The authors believe this amendment ensures the analysis is both methodologically sound and clinically meaningful. Moreover, a modified intention-to-treat approach is well-established in clinical trials where eligibility criteria depend on post-randomisation diagnostic clarification.

The original article was corrected.
